# Supplementary material for: MANIPULATING MITOCHONDRIAL REACTIVE OXYGEN SPECIES ALTERS SURVIVAL IN UNEXPECTED WAYS IN A DROSOPHILA MODEL OF NEURODEGENERATION
Source: bioRxiv. 2024 Mar 29:2024.03.25.586603. Preprint. [Version 1] doi: 10.1101/2024.03.25.586603 (PMC10996551; doi:10.1101/2024.03.25.586603)
Supplement: Supplement 2 [file NIHPP2024.03.25.586603v1-supplement-2.pdf]

bioRxiv preprint doi: https://doi.org/10.1101/2024.03.25.586603; this version posted March 29, 2024. The copyright holder for this preprint (which was not certified by peer review) is the author/funder. This article is a US Government work. It is not subject to copyright under 17 USC 105 and is also made available for use under a CC0 license.

SUPPLEMENTAL FIGURES AND TABLES.

Supplementary Figure 1

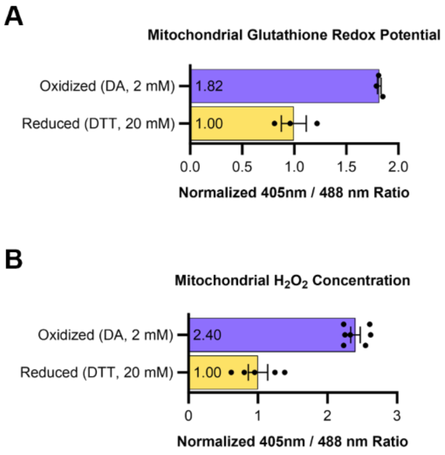

Supplementary Figure 1. mito-roGFP2 biosensors capture redox changes in the *Drosophila* mushroom body. (A-B) Oxidation (with DA) or reduction (with DTT) of 10 day-old WT flies expressing *201Y>mito-roGFP2-Grx1* (A) or *201Y>mito-roGFP2-Orp1* (B) measuring the mitochondrial glutathione redox potential or the H_2O_2 concentration, respectively. 405nm/488nm ratios have been normalized by setting the average reduced 405nm/488nm ratio to 1. A higher 405nm/488nm ratio indicates a more oxidized mitochondrial glutathione redox potential (A) or a higher mitochondrial H_2O_2 concentration (B).

bioRxiv preprint doi: https://doi.org/10.1101/2024.03.25.586603; this version posted March 29, 2024. The copyright holder for this preprint (which was not certified by peer review) is the author/funder. This article is a US Government work. It is not subject to copyright under 17 USC 105 and is also made available for use under a CC0 license.

Supplementary Figure 2

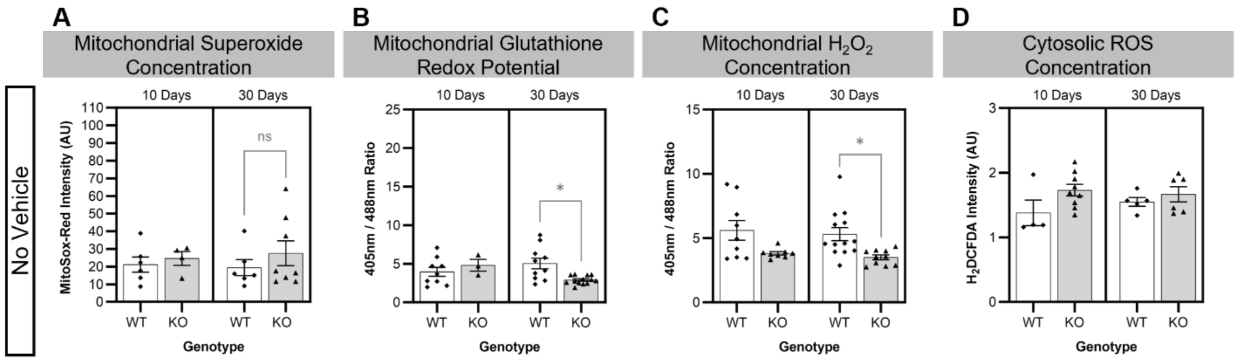

Supplementary Figure 2. WT and Cdk5α-KO flies raised on Caltech media and not exposed to vehicle show similar changes in redox parameters to flies exposed to vehicle.
(A) Quantification of mitochondrial superoxide concentration in WT or Cdk5α-KO brains. A higher MitoSox-Red intensity indicates a higher mitochondrial superoxide concentration. *p* values: * < .05; ** < .01, *** < .001, **** < .0001.
(B) Quantification of mitochondrial glutathione redox potential in WT or Cdk5α-KO MBs. A larger 405nm/488nm ratio indicates a more oxidized mitochondrial glutathione redox potential. *p* values: * < .05; ** < .01, *** < .001, **** < .0001.
(B) Quantification of mitochondrial H_2O_2 concentration in WT or Cdk5α-KO MBs. A larger 405nm/488nm ratio indicates a higher mitochondrial H_2O_2 concentration. *p* values: * < .05; ** < .01, *** < .001, **** < .0001.
(D) Quantification of normalized H_2DCFDA in WT or Cdk5α-KO brains. A higher H2DCFDA intensity indicates a higher cytosolic ROS concentration. *p* values: * < .05; ** < .01, *** < .001, **** < .0001.

bioRxiv preprint doi: https://doi.org/10.1101/2024.03.25.586603; this version posted March 29, 2024. The copyright holder for this preprint (which was not certified by peer review) is the author/funder. This article is a US Government work. It is not subject to copyright under 17 USC 105 and is also made available for use under a CC0 license.

<u>*Supplementary Figure 3*</u>

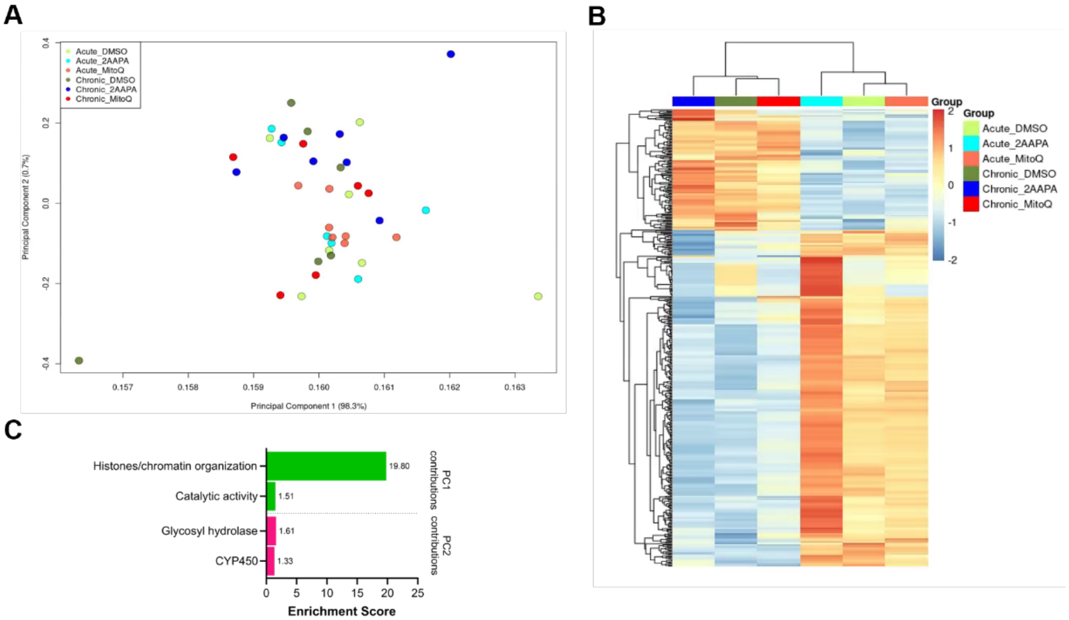

Supplementary Figure 3. Transcriptomic profiling of acute and chronically fed 10 day-old WT flies reveals changes associated with vehicle treatment between acute and chronic groups, but not among drug treatments.
(A) PCA plot of 10 day-old WT flies fed either DMSO (vehicle), 2-AAPA, or mitoQ acutely or chronically.
(B) Bar graph showing the enrichment score of the functional annotation clusters calculated by DAVID 6.8 of 184 differentially expressed genes that contribute significantly to PC1 and PC2 (Huang et al., 2009; Sherman et al., 2022).
(C) Gene heatmap of 184 differentially expressed genes that contribute significantly to PC1 and PC2. Selected genes had a linear fold change of means > |1.5| and a corrected $p < .05$.

bioRxiv preprint doi: https://doi.org/10.1101/2024.03.25.586603; this version posted March 29, 2024. The copyright holder for this preprint (which was not certified by peer review) is the author/funder. This article is a US Government work. It is not subject to copyright under 17 USC 105 and is also made available for use under a CC0 license.

Supplementary Table 1

Drug Abbreviation	Drug Name	CAS Number	Supplier	Catalog Number	Vehicle Solvent	Drug Concentration
**2-AAPA**		1133387-90-2	Sigma-Aldrich	A4111	DMSO	10 µM
**3-AT**	3-amino-1,2,4-triazole	61-82-5	Sigma-Aldrich	A8056	H2O	500 µM
**Antimycin A**		1397-94-0	Sigma-Aldrich	A8674	EtOH	9.11 µM
**Auranofin**		34031-32-8	Sigma-Aldrich	A6733	DMSO	1 µM
**BSO**	Buthionine sulfoximine	83730-53-4	Cayman Chemical	14484	H2O	10 mM
**DDC**	Diethyldithiocarbamate	20624-25-3	Sigma-Aldrich	D3506	H2O	1 mM
**EUK-8**		53177-12-1	Sigma-Aldrich	SML0742	DMSO	500 µM
**Idebenone**		58186-27-9	Sigma-Aldrich	I5659	DMSO	10 µM
**IM**	Imisopasem manganese	218791-21-0	MedChemExpress	HY-13336	DMSO	500 µM
**mitoQ**	Mitquinone mesylate	845959-50-4	Cayman Chemical	29317	EtOH	100 µM
**mtTEMPO**	Mito-TEMPO	1334850-99-5	Sigma-Aldrich	SML0737	EtOH	1 mM
**NAC**	N-acetyl cysteine	616-91-1	Sigma-Aldrich	A7250	DMSO	1 mM
**PQ**	Paraquat	1910-42-5	Sigma-Aldrich	36541	H2O	100 µM
**Rotenone**		83-79-4	Sigma-Aldrich	R8875	DMSO	10 µM
**S1QEL1.1**		897613-29-5	Cayman Chemical	20982	DMSO	800 nM
**S3QEL2**		890888-12-7	Cayman Chemical	18556	DMSO	800 nM

Supplementary Table 1. Summary of drugs used in this study.
Drugs used in this study with CAS identification number, supplier information, solvents and concentrations used to prepare drug for feeding. For non-standard drug abbreviations, a full drug name is included if one exists.

bioRxiv preprint doi: https://doi.org/10.1101/2024.03.25.586603; this version posted March 29, 2024. The copyright holder for this preprint (which was not certified by peer review) is the author/funder. This article is a US Government work. It is not subject to copyright under 17 USC 105 and is also made available for use under a CC0 license.

Supplementary Table 2

Drug	Target of Action	Effect on Target	Survival	Superoxide	Glutathione	H_2O_2
**2-AAPA**	TrxR & GR	Inhibitor	↓	–	↑	–
**3-AT**	Catalase	Inhibitor	↓	–	–	↑
**Antimycin A**	ETC complex III	Inhibitor	↓	↑	↑	↑
**Auranofin**	TrxR	Inhibitor	↓	–	↑	–
**BSO**	GCL	Inhibitor	↓	–	↓	–
**DDC**	Cu/Zn-SOD	Inhibitor	↓	↑	↓	↓
**EUK 8**	Mn-SOD & catalase	Mimetic	↑	↓	–	↑
**Idebenone**	CoQ(10)	Mimetic	↑	↓	–	↓
**IM**	Mn-SOD	Mimetic	↑	↓	–	↑
**mitoQ**	CoQ(10)	Mimetic	↑	↓	–	↓
**mtTEMPO**	Free radicals	Scavenger	↑	↓	–	–
**NAC**	Glutathione	Precursor	↑	–	–	↑
**Paraquat**	Free radicals	Toxicant	↓	↑	↑	↑
**Rotenone**	ETC complex I	Inhibitor	↓	↑	↑	↑
**S1QEL1.1**	ETC complex I	Suppressor of electron leak	↑	↓	–	↓
**S3QEL2**	ETC complex III	Suppressor of electron leak	↑	↓	–	↓

Key	
↑ (green)	**Improves** survival
↓ (magenta)	**Reduces** survival
↑ (orange)	**Increases** oxidation state
↓ (blue)	**Decreases** oxidation state
–	**No change**

Supplementary Table 2. Summary of drugs used in acute drug administration screen and their predicted effects. Drugs used in acute drug administration screen with their mechanisms of action and their predicted effects on survival, mitochondrial superoxide concentration, mitochondrial glutathione redox potential, and mitochondrial H_2O_2 concentration. The predicted drug effects are based on their mechanisms of action as well as experimental evidence in various *in vitro* and *in vivo* contexts. The predicted drug effects are indicated by the green up arrow (drug predicted to improve survival), the magenta down arrow (drug predicted to reduce survival), the orange up arrow (drug predicted to lead to an increased oxidation state), the blue down arrow (drug predicted to lead to a decreased oxidation state), or a black bar (drug predicted to led to no change in survival or oxidation state or a prediction cannot be made for the given drug).

bioRxiv preprint doi: https://doi.org/10.1101/2024.03.25.586603; this version posted March 29, 2024. The copyright holder for this preprint (which was not certified by peer review) is the author/funder. This article is a US Government work. It is not subject to copyright under 17 USC 105 and is also made available for use under a CC0 license.

Supplementary Table 3

See "Wodrich et al. – Supplementary Data.xlsx"

Supplementary Table 3. Data presented in this study.
Summary data presented in this study, excluding raw RNA sequencing data (see above).
